# Bilateral simultaneous rupture of the quadriceps tendon in a patient with psoriasis: a case report and review of the literature

**DOI:** 10.1186/1752-1947-5-331

**Published:** 2011-07-29

**Authors:** Shanaka Senevirathna, Sarkell Radha, Aysha Rajeev

**Affiliations:** 1 Department of Trauma & Orthopaedics, Queen Elizabeth Hospital, Gateshead, NE9 6SX, UK

## Abstract

**Introduction:**

Bilateral quadriceps tendon rupture is not common in the absence of systemic disease. Patients with chronic systemic diseases such as uremia and systemic lupus erythematosus and patients who are being treated with systemic steroids or local steroid injections are more prone to tendon rupture. The tendon can rupture spontaneously or as a result of trauma. We report an unusual case of simultaneous bilateral traumatic quadriceps tendon rupture in a patient with psoriasis who was being treated with topical steroid preparations.

**Case presentation:**

A 57-year-old Caucasian man with a known history of psoriasis, for which he was being treated with topical steroid preparations, presented to our hospital with clinical signs of bilateral quadriceps tendon rupture after he fell while walking down stairs. The diagnosis was confirmed by bilateral ultrasound scans of the thighs. The patient underwent surgery to repair both quadriceps tendons. Post-operatively, the patient was immobilized first in bilateral cylinder casts for six weeks, then in knee braces for the next four weeks. His knees were actively mobilized during physiotherapy.

**Conclusion:**

Bilateral quadriceps tendon rupture is a rare occurrence in patients with psoriasis who are being treated with topical steroids.

## Introduction

Bilateral quadriceps tendon rupture is extremely rare in the absence of systemic disease. The co-existence of systemic and local disease is taken into consideration in the pathogenesis of these ruptures. The pre-disposing factors for spontaneous tendon rupture include chronic systemic disease, treatment with systemic steroids or local steroid injections, or trauma [1-4]. In the present report, we describe a rare case of simultaneous bilateral traumatic quadriceps tendon rupture in a patient with psoriasis who was being treated only with topical steroid preparations and was not taking systemic steroids.

## Case report

A 57-year-old Caucasian man with a history of psoriasis, for which he was taking topical steroid preparations, fell while walking down stairs. Initially, his left leg gave way, and he landed on his hyperflexed right knee. He had been unable to bear weight on his legs since then and presented to our Accident and Emergency Department with painful swelling over both knees.

His physical examination revealed that both knees were very tender to touch over the suprapatellar region and had massive suprapatellar swelling. He was unable to perform a straight leg raise on both sides, although active quadriceps contraction was seen. On palpation, a defect in the continuity of both quadriceps tendons was found.

**Figure 1:**
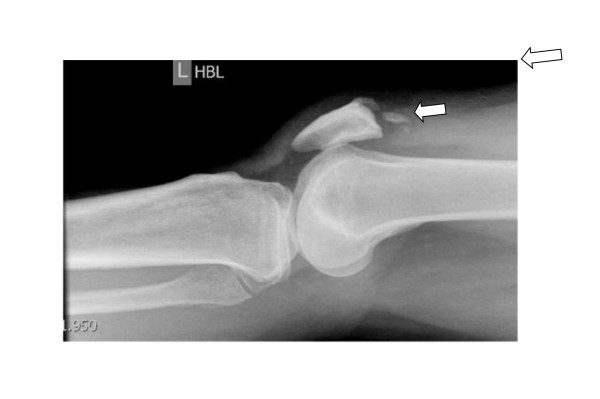
**Radiograph of the left knee showing quadriceps rupture**.

Plain radiographs of both knees revealed joint effusion, patella baja, and disruption of soft tissues superior to the patella. An avulsion fracture of the patella on the left side was suspected (Figure 1). Bilateral ultrasound scans of the thighs confirmed the diagnosis of bilateral quadriceps tendon rupture at the osseotendinous junctions and a calcified fragment indicating a possible avulsion fracture within the detached end of the left quadriceps tendon.

**Figure 2:**
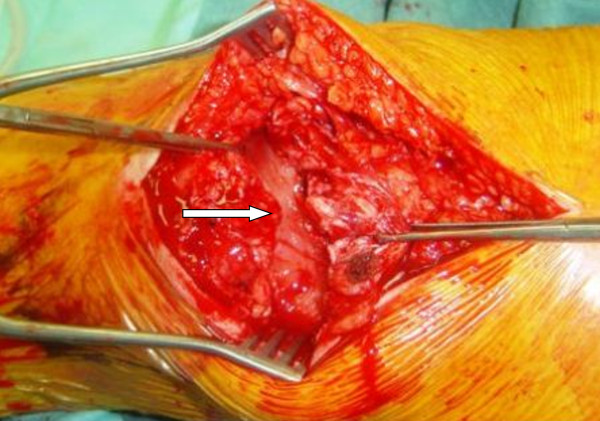
**Intra-operative image showing quadriceps rupture**.

The patient underwent bilateral surgical exploration of the knees with longitudinal incisions. The peri-operative findings were complete rupture of the tendons bilaterally from the superior pole of the patella (Figure 2). Bilateral quadriceps surgical repairs were performed using mid-line incisions over the knees, and the ruptured ends of the quadriceps tendons were identified. The ruptured ends were freshened and repaired using Vicryl 2-0 sutures through drill holes in the patella to the tendons. Medial and lateral retinacular repair was performed using Vicryl 2-0 and 1-0 sutures.

**Figure 3:**
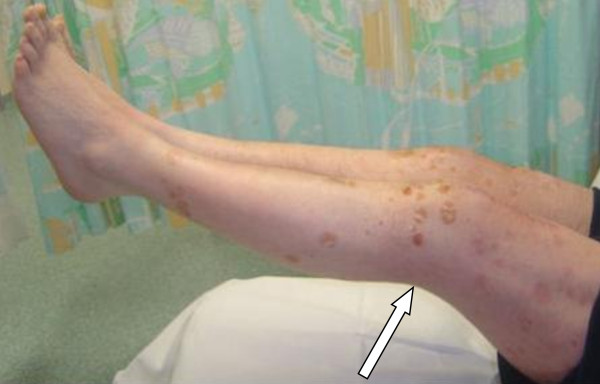
**Image obtained three months after surgery showing full, straight leg raise using both knees**.

Post-operatively, the patient was immobilized first in bilateral cylinder casts for six weeks, then in knee braces for the next four weeks. His knees were actively mobilized during physiotherapy. The physiotherapy protocol was initially active knee range of motion exercises, which were followed by passive assisted and polymeric exercises. The patient had an uneventful post-operative recovery and was able to perform straight leg raises without a lag by the time of his three-month follow-up examination (Figure 3). His final knee range of motion was 0° to 125° in both legs after a full course of physiotherapy. He was discharged from the clinic after six months and returned to work.

## Discussion

Few reports of quadriceps tendon rupture exist in the literature. An older adult patient may present with an inability to walk, and a diagnosis of proximal myopathy usually precedes the true diagnosis of spontaneous rupture. Often the presentation causes diagnostic confusion because of bilateral involvement and the absence of trauma.

Lewis *et al*. [5] reported a case of bilateral quadriceps tendon rupture in a bodybuilder that was attributed to anabolic steroid misuse. Many cases of bilateral quadriceps tendon rupture have been reported in patients with chronic renal failure [1-3].

Bhole *et al*. [4] discussed the mechanisms, variability at the rupture site, pathogenesis, and histopathological changes of quadriceps tendon rupture in patients with uremia. The various systemic diseases that pre-dispose people to quadriceps tendon rupture include rheumatoid arthritis, arteriosclerosis, diabetes mellitus, systemic lupus erythematosus (SLE), primary and secondary hyperparathyroidism, gout, tuberculosis, vasculitis, and steroid injections to the tendons [3,4]. Few cases of simultaneous quadriceps tendon and contralateral patellar tendon rupture have been described in the literature [1,3,6,7].

In their case report, Muratli *et al*. [1] took into consideration mechanical factors and co-existing systemic and local factors associated with quadriceps tendon rupture. The most important factor seems to be the blood supply to the tendon, which comes from the arterioles of the nearby muscles and connective tissue. After micro-trauma, the blood supply to the tendon diminishes because of the infiltration of mononuclear cells and thrombosis of the micro-circulation, and thus the tendon becomes more susceptible to rupture [8].

Anzel *et al*. [9] stated that athletes and laborers are more susceptible to ruptures. Endothelial swelling with peri-vascular lymphocytic exudate has been described in patients with arthritis, and peri-vascular mononuclear cell infiltrate in the peri-vascular area was observed in a patient with SLE. In some patients with SLE who are being treated with corticosteroids, tendon rupture has been observed without any inflammatory reactions [8]. In patients with rheumatoid arthritis, increased levels of collagenase may play a role in the development of tendon degeneration and subsequent rupture [10]. Bilateral quadriceps tendon rupture has also been reported in patients with amyloidosis [11].

Rasul and Fischer [12] reported that isolated quadriceps tendon rupture usually occurs after trauma in the sixth or seventh decade of life. The sites of rupture are classified as the musculotendinous, mid-tendinous, and osseotendinous junctions [13]. Rupture through the substance of the tendon, which is extremely rare, has been reported in a case of glomerulonephritis [14]. Tendon ruptures in patients with chronic renal failure tend to occur at a low activity level and may give the initial impression of being trivial [4].

On the basis of his series of 55 cases of simultaneous bilateral quadriceps tendon ruptures, Shah [2] stated that falls are the main cause (76%) and that the commonest site of rupture is the osseotendinous junction (60%). The patients in his study were almost always treated surgically (96%). According to his report, the patient's gender, mechanism of injury, and tear location, as well as the time to diagnosis and repair, were not related to outcome, whereas the patient's age, multiple risk factors, renal or endocrine disease, or diabetes were related to outcome.

Quadriceps tendon rupture after trauma occurs by direct injury or by the sudden, violent contraction of the muscle against the body weight with the knees in a semi-flexed position in an effort to prevent a fall or to lift something or simply in descending stairs [3,14].

Rogers *et al*. [6] reported a case of quadriceps tendon rupture with contralateral patellar tendon rupture in a 47-year-old healthy man and emphasized the importance of the position of the limb and the degree of knee flexion at the time of injury.

In people younger than 50 to 60 years of age, the patellar tendon is the weakest link in the quadriceps mechanism, fracturing 50 to 60 times more frequently than in other ruptures. Indirect trauma accounts for more ruptures of the quadriceps tendon than direct trauma, and the site of rupture is suprapatellar in two-thirds of patients and infrapatellar in one-third of patients [3].

Arumilli *et al*. [15] reported a case of bilateral simultaneous complete quadriceps tendon rupture in a patient who was being treated for enthesopathy of the quadriceps tendons on both sides. They believe that chronic enthesopathy of the superior pole of patella made their patient's quadriceps tendons susceptible to complete rupture due to eccentric loading. McMaster [13] showed that normal tendons would not normally break, even if half-severed, until the loading profile reached about 10 to 15 kPa/mm^2^, a level at which the belly of the muscle, its osseotendinous insertion, and even the femur would fail.

Lighthart *et al*. [16] compared the biomechanical strength between bone tunnel repair and suture anchors. They found no statistical difference in mean initial displacement after 10 cycles between suture anchor and bone tunnel repairs on the lateral or medial side. They also observed no difference in displacement between the two types of repairs with the patient in a resting position (no load) or in leg extension with load after 1000 cycles.

The rehabilitation protocol following quadriceps tendon repair is more or less standardized. After surgery, the knees are immobilized in extension for six weeks, followed by gradual weight bearing and gait training with the patient in knee braces. The patient is then weaned off the knee braces, and the patient's range of motion is then increased to strengthen the knees [17]. Most reported case series have described good functional outcomes. Approximately two-thirds of patients recovered to the same or better peak torque/body weight ratio, average power, maximum average peak torque, and total work/body weight ratio in affected and unaffected limbs [18]. Most patients who undergo bilateral simultaneous or unilateral tendon repair can expect a good recovery of range of motion and can return to their previous occupation, but many have persistent weakness and difficulty returning to higher-level sporting activities [18,19].

## Conclusion

Simultaneous bilateral quadriceps tendon rupture is a rare occurrence in patients with psoriasis. Our patient was not being treated with systemic steroids or any other medications that would have weakened the quadriceps tendons. Herein we report one of the causes of bilateral quadriceps tendon rupture.

## Consent

Written informed consent was obtained from the patient for publication of this case report and any accompanying images. A copy of the written consent is available for review by the Editor-in-Chief of this journal.

## Competing interests

The authors declare that they have no competing interests.

## Authors' contributions

SS was the main author who wrote the manuscript. SR helped with taking the photographs. AR was the senior author and managed the patient's surgery.
